# Correction to “Essential Thrombocythemia's Role in the Complex Landscape of Vasculitis: A Case Report”

**DOI:** 10.1002/ccr3.71301

**Published:** 2025-10-27

M. R. Jafari Nakhjavani, S.‐T. Mirzohreh, S. Kolahi, et al., “Essential Thrombocythemia's Role in the Complex Landscape of Vasculitis: A Case Report,” *Clinical Case Reports* 13, no. 10 (2025): e70963. https://doi.org/10.1002/ccr3.70963.

The spelling for one of the authors' name—Arezoo Ghasembaglou was corrected in this version as Arezou Ghassembaglou.

We apologize for this error.

